# Low back pain development response to sustained trunk axial twisting

**DOI:** 10.1007/s00586-013-2784-7

**Published:** 2013-04-24

**Authors:** Xinhai Shan, Xiaopeng Ning, Zhentao Chen, Meng Ding, Weifei Shi, Shulong Yang

**Affiliations:** 1 Biomechanics Laboratory, College of Physical Education, Shandong Normal University, 88 Wenhua East Road, Jinan, 250014 Shandong China; 2 Industrial and Management Systems Engineering, West Virginia University, Morgantown, WV 26506-6107 USA

**Keywords:** Flexion relaxation phenomenon, Spinal twist, Rotational creep, Low back pain, Visual analog scale

## Abstract

**Purpose:**

To investigate if there is an effect of sustained trunk axial twisting on the development of low back pain.

**Methods:**

Sixteen male pain-free university students volunteered for this study. The trunk axial twisting was created by a torsion moment of 50 Nm for 10-min duration. The axial rotational creep was estimated by the transverse camera view directly on the top of the head. The visual analog scale in low back area was examined both in the initial and at the end of twisting. Each performed three trials of lumbar flexion–extension with the cycle of 5 s flexion and 5 s extension in standing before and after twisting. Surface electromyography from bilateral erector spinae muscles as well as trunk flexion performance was recorded synchronously in video camera. A one-way ANOVA with repeated measures was used to evaluate the effect of twist.

**Results:**

The results showed that there was a significant (*p* < 0.001) twist creep with rotational angle 10.5° as well as VAS increase with a mean value 45 mm. The erector spinae was active in a larger angle during flexion as well as extension after trunk axial twisting.

**Conclusions:**

Sustained trunk axial twisting elicits significant trunk rotational creep. It causes the visual analog scale to have a significant increase, and causes erector spinae muscles to become active longer during anterior flexion as well as extension, which may be linked to the decrease of the tension ability of passive tissues in low back area, indicating a higher risk in developing low back pain.

## Introduction

Low back pain (LBP) is a serious and complex medical condition with high prevalence rate, compensation cost [1, 2] and long recovery time [3]. In the United States alone, the annual total cost of LBP was estimated from 100 to 200 billion dollars [4]. However, the underlying mechanism of LBP development is still poorly understood.

An early industrial surveillance study investigated workers’ trunk kinematics during the performance of more than 400 repetitive manual material handling industrial jobs. Their results discovered that the occurrence of pain and disorders in the lower back region is strongly associated with trunk axial twisting [5]. Recent in vivo study demonstrated that the increase of inter-facet spacing may reduce the twisting stiffness of lumbar motion segment and increase the trunk twisting range of motion (ROM) [6]. Because the size of the facet joint is relatively small compared to the amount of force it undertakes during trunk twisting, this joint is prone to degeneration which may lead to LBP [7].

Historically, a number of in vitro studies investigated the injury mechanism of trunk twisting motion. An early study examined 66 human lumbar spine specimens and concluded that lumbar intervertebral disc (IVD) injury and degeneration could be caused by vertebrae axial rotation [8]. Human cadaver study found that during lumbar axial twisting motion, facet joint serves as a critical component which limits rotation boundary as well as excessive shear forces and moment [9]. In a more recent cadaver study, researchers found that the reduction of gap between articulating surfaces of the facet joints significantly reduces the lumbar twisting ROM [10]. In addition to the human cadaver studies, animal model has also been studies. In 2005, Drake et al. [11] evaluated the effect of prolonged axial loading on the failure mechanics of porcine cervical motion segments during cyclic sagittal flexion–extension motion. The results of this study revealed that the axial torque increases the chance of facet joint fracture and IVD herniation. Given the similarity between porcine cervical spine and human lumbar spine [12], these results could be used as strong yet indirect evidence that links repetitive lumbar axial loading and LBP upon human.

Although trunk twisting has been identified as a major factor that could contribute to LBP, previous investigation of lumbar passive tissue creep has been focused only in the sagittal plane. One study investigated the effect of prolonged sagittal symmetric bending on the load sharing mechanism between lumbar active (muscles) and passive (facet capsule, ligaments, fascia and IVD) tissues [13]. Results of that study discovered that the shift of external loading from active tissue to passive tissue was delayed due to lumbar passive tissue creep. Previous in vivo study discovered the exponential relationship between the lumbar twisting angle and passive resistance [14]. Authors of that study indicated that the elastic forces generated by the passive component of muscles are the main sources of passive resistance at the initial twisting motion, and then toward the end of ROM lumbar posterior ligaments and IVD will start to generate elastic forces and become the main contributor. This finding suggests that prolonged trunk axial twisting could also generate passive tissue creep and cause an alternation in the synergy between lumbar active and passive tissues.

The synergy between lumbar active and passive tissues during trunk flexion–extension motion represents the load sharing mechanisms in the lumbar region, and the flexion relaxation phenomenon (FRP) could be used to reveal the critical characteristics of this mechanism. FRP describes the cessation [including electromyography (EMG) silence during flexion and EMG initiation during extension] of posterior lumbar muscle EMG activity at close to full flexion posture [15–17]. Combining with visual analog scale (VAS), a self-rating of current level of perceived pain [18, 19], the change of FRP response could be utilized as a reference to evaluate LBP development [16, 17].

Previous research revealed that, LBP development could be elicited by prolonged lumbar flexion [17], by prolonged standing [18, 20] and/or by sustained spinal compressing [16]. However, there are limited evidences regarding the effect of sustained trunk axial twisting on LBP development.

Therefore, the purpose of the study was to investigate the effect of sustained trunk axial twisting on LBP development. It was hypothesized that sustained trunk axial twisting would elicit a significant trunk axial rotational creep and a significant increase of VAS score. It was also hypothesized that FRP response would be changed both in EMG silence during flexion and in EMG initiation during extension after sustained trunk axial twisting.

## Materials and methods

### Subjects

Sixteen male subjects were recruited from the University student population to participate in the study which was approved by local ethical committee. Subjects read and signed a consent form before participating in the study. Demographic information was collected using a questionnaire to screen for inclusion and exclusion criteria. Their age, weight, height, and BMI index (mean (SD) [minimum–maximum]) were 23(2) [19–25] years, 73(6) [61–85] kg, 178(5) [170–190] cm, 23(2) [20–25] kg/m^2^, respectively. Subjects without current complaints of back pain were included in the study. Exclusion criteria consisted of any uncorrectable spine pathology, history of spine surgery, current neurological disorder, hip conditions that would not allow the subjects to fully flex and extend their hips comfortably, current back pain, consultation of a physician for back pain in the last year.

### Twist creep measurement

**Fig. 1:**
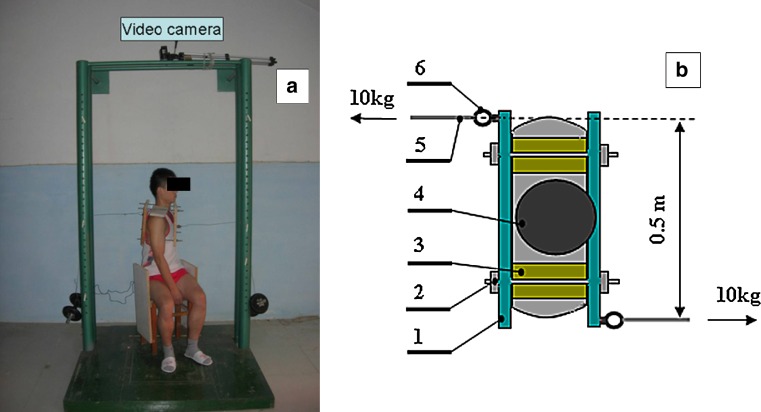
A subject in trunk axial twisting period. **a** *Front view* of the subject, **b** *top view* of the subject. *1* wooden plate, *2* screw, *3* soft cushion, *4* subject’s head, *5* steal cable, *6* hook

A special stool was designed to restrict the rotation of hip and thigh in the sitting position during trunk axial twisting (Fig. 1a). A wooden frame, which includes two plates and four screws, was designed to clamp the individual’s rib cage. The width of the frame could be adjusted by screw to fit the anterior–posterior thickness of the trunk in thoracic lever. This frame could be put on subject’s shoulder through two upper screws with soft cushions. The total weight of the frame is about 1.5 kg.

Two hooks were fixed on the edge of each plate (Fig. 1b). One 10-kg barbell was connected to each hook with a steel cable. The weight of the barbells created two horizontal forces with opposite directions, which generated a near constant twisting torque of 50 Nm with regard to the trunk center of rotation. (Fig. 1a, b).

The sustained trunk twisting lasted for 10 min. Before twisting, subject was required to twist to the left as much as possible. Barbells were then hooked and the steel cables were adjusted to maintain horizontal and perpendicular to the wooden plates as much as possible. The subject was required to keep relax during 10-min twisting.

Rotational angle, or trunk twist creep, was estimated by a digital camera (Panasonic-SDR-H85) fixed on the top of the rigid barbell frame about 1 m above the subject’s head (Fig. 1a). The camera collected kinematic data at the rate of 50 frames per second.

### Visual analog scale examination

VAS is measured on a linear scale in millimeters in vertical style. A VAS of zero is relative to no pain, and a VAS of 100 to unbearable pain.

Participants rated their level of neck, shoulder, upper back, and low back pain on a 100 mm VAS in two separate times: prior to the start and at the end of trunk twisting. For this study, we considered the LBP rating only as it was found to be the most consistently increased body area [18].

### Flexion relaxation measurements

The pre-gelled (Ag–AgCl) disposable surface electromyography electrodes were applied at the L3–4 level over the erector spinae (ES) musculature (about 4–6 cm lateral from midline) bilaterally. Inter-electrode distance was 2.5 cm, and the electrodes were oriented longitudinally along the muscle. A reference electrode was placed on the left anterior superior iliac crest. The EMG signals were amplified ×1,000 with a frequency bandpass of 10–500 Hz, 1 μV noise referred to input, and CMRR of 120 dB. The Input impedance was 10^9^ kΩ. The resulting signal was sampled at 1,000 Hz via a 14-bit data acquisition system and stored for later processing.

Angular variables during the performance of anterior flexion–extension were estimated by another digital camera (Panasonic-SDR-H85) placed 3 m away from the subject at waist level with a direct view of the subject’s right side in the sagittal plane. The camera collected kinematic data at the rate of 50 frames per second.

Three circular markers used to measure inter-segment angles were attached to the subjects as follows: right lateral greater trochanter, lateral midline along the iliac crest, lower palpable edge of the rib cage [17]. The set-up of markers allows calculations of the angles relative to variation of lumbar flexion.

Video and EMG data were synchronized by a light emitting diode which turned on and off at the same time as the recording of EMG signals.

### Protocol

The skin was cleansed and lightly abraded with alcohol prep pads before EMG electrode attachment. The electrodes and skin markers were placed as described above, and a signal check was performed to ensure the quality of EMG signals, and clear markers on the video.

Before twisting, maximal voluntary contractions (MVC) were obtained for left and right ES through applying resistance in the Beiring–Sorensen position [20, 21].

After finishing MVC test, the subject was then required to perform flexion–extension in standing. During the performance, each subject was required to put the feet shoulder width apart, and keep the knee straight during the test [16, 22], and make fingernails contacting toes of feet in full flexion. Each trial consisted of 10 s total time: 5 s from upright posture to full anterior flexion and 5 s from full flexion back to upright posture [16, 23]. The timing for each trial was set by a metronome with one beat per second. Each subject performed three full flexion trials, with 30 s rest between them.

After finishing three full flexion trials, subject was then required to perform sustained trunk twisting task as described above (Fig. 1). During twisting, the performance was recorded by video camera in direct transverse view for 10 min. The VAS was examined both at the beginning and at ending of twisting. Upon the finish of sustained twisting task, three anterior flexion–extension performances were tested again in the same way.

### Analysis

The video data both from transverse and sagittal view were digitized and transformed to two-dimensional space using the APAS (Ariel Performance Analysis System, USA) software. The Kinematic data were smoothed using a zero lag fourth-order Butterworth digital low-pass filter with a cutoff frequency of 1 Hz.

For the trunk axial rotation angle, two points were selected on the wooden plate to represent a line in a direct view of the transverse plane. To eliminate the effect of elastic strain [14, 17], the beginning of the creep was defined as 5 s after the load being applied. The duration from the beginning to the end of twist was set to 10 min. The average angle of the line in ten continuous video frames at the beginning and the end point was set to be the initial angle (normalized to be zero) and the rotational angle (twist creep), respectively.

For FRP in anterior flexion–extension, two angles suggested by Solomonow et al. [17] were considered to be of interest: the angle of trunk inclination, *α*, defined as the angle between the line of two markers (lateral midline along the iliac crest, the lower palpable edge of the rib cage) and the vertical line to ground through the marker on the iliac crest and the angle of lumbar flexion, *β*, defined as the angle of trunk inclination minus the hip flexion angle (defined as the angle between the vertical line crossing the ilium marker and the line connecting the greater trochanter and ilium markers). Subsequently, flexion refers to the angle representative of lumbar flexion, and inclination refers to the trunk inclination angle relative to the ground.

**Fig. 2:**
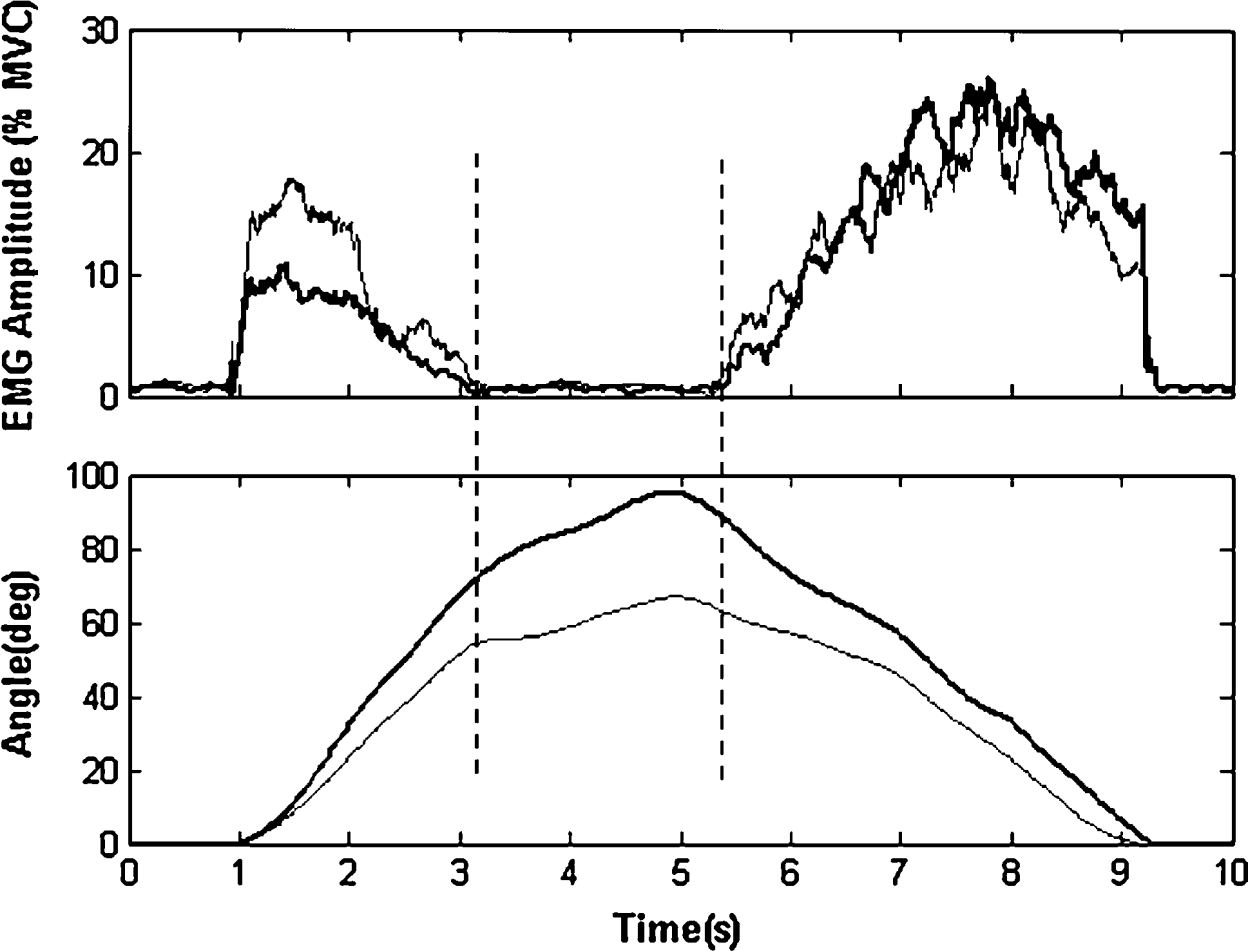
The exemplar data of bilateral EMG amplitude (%MVC) and the recorded angles. *Top* the EMG-On *right* ES (*thick*) as well as *left* ES (*thin*), *Lower* the recorded angles of trunk inclination (*thick*) and lumbar flexion (*thin*). The *parallel vertical lines* represent EMG-On and -Off timing

EMG signals had systematic bias removed, and were full wave rectified prior to being dual pass filtered through a fourth-order Butterworth filter with an effective cutoff frequency of 6 Hz [24]. The resulting linear envelope signals were then normalized to MVC to obtain normalized EMG (% MVC) (Fig. 2). Then, the normalized EMG from bilateral ES muscles was averaged to represent the bilateral ES muscle activations [25].

A threshold level, 1 % of MVC was used to initially determine the beginning and the ending of the myoelectric activity [26]. Only 12 subjects were selected to be the statistical samples as they met the strict threshold during flexion relaxation period in the performance of anterior flexion–extension both before and after twisting.

The following parameters were determined according to the synchronized time: maximum angle (the angle at the full flexion position, including flexion angle and inclination angle); “EMG-Off” angle (the angle at the position where EMG silence begins during trunk flexion); “EMG-On” angle (the angle at the position where EMG activity begins during trunk extension to the initial standing position); the normalized angle defined by the angle relative to the percentage of the maximum angle. All these parameters are shown in Table 1.

**Table 1:** Results of normalized data and statistical analysis (*n* = 12)

Parameters (°)	Absolute mean (SD)		*p* value	Normalized mean (SD)		*p* value
	Before twist	After twist	Effect of twisting	Before twist	After twist	Effect of twisting
Flexion angle	62.0 (9.3)	64 (10.5)	0.632	100.0 (.0)	100.0 (0.0)	–
EMG-Off flexion	41.5 (9.4)	53.1 (7.2)	0.003**	67.5 (15.5)	83.4 (7.1)	0.004**
EMG-On flexion	50.4 (6.0)	56.4 (8.8)	0.065	81.9 (8.3)	88.5 (9.0)	0.074
Inclination angle	92.8 (11.9)	91.5 (11.8)	0.797	100.0 (0.0)	100.0 (0.0)	–
EMG-Off inclination	59.1 (10.0)	68.5 (10.2)	0.035*	64.8 (13.9)	75.3 (9.9)	0.044*
EMG-On inclination	66.7 (9.5)	77.0 (12.9)	0.036*	72.5 (10.0)	84.2 (9.5)	0.007**
Rotational angle (*n* = 16)	0.0 (0.0)	10.5 (4.2)	<0.001**	–	–	–
VAS (mm) (*n* = 16)	3.3(5.0)	48.4 (28.2)	<0.001**			

* *p* < 0.05, ** *p* < 0.01; *VAS* visual analog scale

A one-way ANOVA with repeated measures was used to evaluate the effect of twisting. The alpha level was set at 0.05.

## Results

The result showed that after 10-min static axial spinal twisting with 50 Nm twisting moment, there were significant changes (*p* < 0.001) both on axial spinal twist creep 10.5 (4.2)° and on VAS index 45.1(26.0) (Table 1).

Significant changes were found (*n* = 12) in flexion period both at EMG-Off flexion and at EMG-Off inclination after twisting. At EMG-Off flexion, the absolute value increased significantly (*p* = 0.003) from 41.5° before to 53.1° after whereas the normalized value increased significantly (*p* = 0.004) from 67.5 % before to 83.4 % after twisting. At EMG-Off inclination, the absolute value increased significantly (*p* = 0.0035) from 59.0° before to 68.5° after whereas the normalized value increased significantly (*p* = 0.044) from 64.8 % before to 75.3 % after twisting.

Obvious increases were also found (*n* = 12) in the extension period both at EMG-On flexion and EMG-On inclination. At EMG-On flexion, though not significantly, the absolute value increased obviously (*p* = 0.065) from 50.4° before to 56.4° after whereas the normalized value increased obviously (*p* = 0.074) from 81.9 % before to 88.5 % after twisting. At EMG-On inclination, the absolute value increased significantly (*p* = 0.036) from 66.7° before to 77.0° after whereas the normalized value increased significantly (*p* = 0.007) from 72.5 % before to 84.2 % after twisting.

No significant difference was found (*n* = 12) at either flexion angle (*p* = 0.632) or inclination angle (*p* = 0.797) after twisting.

## Discussion

The major results of this study pointed out that sustained trunk twisting elicits significant spinal rotational creep, and causes significant changes in both VAS and the responses of FRP. Sustained twisting causes an individual to have a large amount of increases in perceived pain and causes erector spinae muscles to become active longer during anterior flexion as well as extension.

Significant increase of trunk axial rotational angle (*p* < 0.001) was found with the value 10.5° as we hypothesized, indicating that significant creep on lumbar passive tissues (e.g., facet capsule, dorsolumbar fascia and posterior ligaments) after 10-min trunk axial twisting, just like the creep elicited by sustained lumbar flexion [17], or by prolonged spinal compressing [16].

Significant increase (*p* < 0.001) was also found in VAS after 10-min trunk twisting [48.4 (28.2) mm after versus 3.3(5.0) mm before]. Since VAS has been found to have a good validity [27] as well as reliability [28], it was suggested by Kelly [29] that 9 mm, the minimum clinically significant difference in VAS, represents a small treatment effect whereas greater than 20 mm represents a large treatment effect. Therefore, 10 mm could be used as a threshold of actually pain [18]. In current study, although the duration of twisting was only 10 min, the VAS increased about 45 mm which was much larger than that elicited through 2-h standing [18, 20], indicating the development of LBP.

The data and the statistical analysis showed that there were significant changes on FRP response during flexion as well as extension after 10 min of trunk twisting. EMG-Off (signal silence) became significantly later during the flexion phase (64.8 % (13.9) versus 75.3 % (9.9), *p* = 0.044, in normalized EMG-Off inclination; 67.5 % (15.5) versus 83.4 % (7.1), *p* = 0.004, in normalized EMG-Off flexion), while EMG-On got earlier on back muscles during extension phase (72.5 (10.0) versus 84.2 (9.5), *p* = 0.007 in normalized EMG-On inclination; 81.9 (8.3) versus 88.5 (9.0), *p* = .074, in normalized EMG-On flexion) .

FRP could be explained as a synergistic load sharing between ES muscles and the viscoelastic elements of lumbar spine. The tension in stretching passive tissues (facet capsule, dorsolumbar fascia and posterior ligaments) in human allows paraspinal muscles to decrease activity [15, 17]. Therefore, the significant change of FRP response may reflect that the tension in posterior passive tissues is below the required force to support the trunk at an earlier flexion angle, and requires the later diminish during flexion phase and earlier EMG activation of active tissues to support the load during extension phase, indicating the development of LBP [16, 17].

It is not clear which of the viscoelastic tissues were active and underwent creep in this investigation. The facet capsule may be one of major tissues, since facet joint is thought to be served as a critical component to resist the shear torsion during trunk axial twisting [7, 9, 11]. Each of other tissues, such as dorsolumbar fascia, posterior ligaments, supraspinous and intraspinous ligaments, is probably one of active tissues in the FRP response and probably is subjected to creep as well [17, 30, 31]. Another important tissue may be the IVD [11, 14]. The shear forces and moment [9] created by spinal twisting within discs might elicit a shrinkage on spine by making the nucleus pulposus loose some fluid just like twisting a cloth full of water. Moreover, spinal shrinkage itself could indeed elicit changes in FRP response according to our recent investigation [16].

Some limitations exist in present study. First, there is no consideration about gender because of the technique of clamping the rib cage in vivo for female individuals. In fact, females are thought to develop slightly more creep than males over the same loading period [17, 32]. Thus, females may have a larger LBP development response to the same protocol of sustained trunk twisting. Secondly, the direction of force caused by the weight of barbell relative to wooden plate may be changed during twisting, making the magnitude of torque being decreased in some degree because of axial rotation (Fig. 1b). However, with a characteristic of symmetry, two forces caused by barbell weights would be always parallel, allowing the trunk have only a performance of twisting. In addition, this effect is within a controllable range because of relative less rotation angle (about 10°). Thirdly, only EMG signals from the superficial erector spinae muscle fibers were recorded in this study. There were no EMG data from deeper muscles such as the multifidus, which may potentially show different responses. Fourthly, the twist torque (about 50 Nm) and duration (10 min) tested in the current study are moderate by comparison with some occupational twisting activities [5, 14]. However, since large changes in VAS and FRP responses were found in this moderate laboratory condition, greater changes in VAS and FRP responses could be expected in more severe twisting conditions.

The general conclusion drawn from the results of this research confirms that sustained trunk twisting elicits significant trunk rotational creep. It causes an individual to have a significant change in VAS in the low back area, and causes erector spinae muscles to become active longer during anterior flexion as well as extension, which may be linked to the decrease of the tension ability of passive tissues in low back area, indicating a higher risk in developing LBP.
